# Bilateral patellar tendon rupture on lupus undergoing corticosteroids: a case report

**DOI:** 10.1186/s12891-020-03513-w

**Published:** 2020-07-21

**Authors:** Zakaria El Ouali, Kawtar Nassar, Elie Bassa, Reda Taoussi, Mouna Sabiri, Mohamed Rafai, Saadia Janani

**Affiliations:** 1 Department of Rheumatology, University Hospital of Ibn Rochd, Casablanca (Morocco), 72 rue Abou Dabi, Oasis, 20410 Casablanca, Morocco; 2 Department of Central Radiology, University Hospital of Ibn Rochd, Casablanca, Morocco; 3 Department of Traumatology and Orthopedics P32, University Hospital of Ibn Rochd, Casablanca, Morocco

**Keywords:** Systemic lupus Erythematosus, Tendon rupture, Corticosteroids, Patella Alta, Ultrasound, Case report

## Abstract

**Background:**

Patellar tendon rupture is a rare condition, especially when it is bilateral. The most frequent associated pathologies are systemic lupus erythematosus, chronic renal failure, or treatments like corticosteroids. The aim of this case report is to draw attention to the non-specific clinical aspect of this condition, to recall its radiological signs, and highlight the diagnostic contribution of musculoskeletal ultrasound.

**Case presentation:**

A 39-year-old man was diagnosed with a systemic lupus erythematosus with cutaneous, pulmonary, cardiac, hematological, renal, and immunological manifestations. He was treated with high-dose corticosteroids. Within 3 months he presented with a total functional impotence of the knees. On physical examination, there was a gap in the right infrapatellar region, his patellae were abnormally ascended, and his left knee was swollen. Insall-Salvati ratio on knees conventional radiographies was 2.5 in the right and 2.25 in the left knee, assessing bilateral patella alta. Ultrasound revealed a complete and bilateral patellar tendon rupture. The treatment consisted in a surgical repair and physiotherapy. The patient was able to mobilize independently after 6 months.

**Conclusions:**

Bilateral patellar tendon rupture is exceptional. Systemic lupus erythematosus and corticosteroids are among trigger factors. Careful examination of the patellae should be done in front of knee extension deficit. Ultrasound plays a determining role in the diagnosis.

## Background

Patellar tendon rupture is a rare condition, tending to result from an overall weakened tendon placed under high tensile forces [1]. Known risk factors are inflammatory rheumatic and systemic diseases, diabetes mellitus, renal dialysis, and treatments like corticosteroids (CS) and fluoroquinolones (FQ) [1]. Simultaneous bilateral patellar tendon rupture (BPTR) is even more sporadically reported, making it more difficult to individualize its contributing factors [2]. We report a case of simultaneous BPTR in a 39-year-old man with SLE undergoing CS. Our purpose is to draw attention to the non-specific clinical aspects of this condition, to recall its radiological signs, and to highlight the diagnostic contribution of musculoskeletal ultrasound (MSUS).

## Case presentation

A 39-year-old man was diagnosed in March 2019 with a SLE following the criteria of the Systemic Lupus International Collaborating Clinics (SLICC), with multi-organ involvement. The cutaneous manifestations included a malar rash, nonscarring alopecia, photosensitivity, and a lupus strip on direct immunofluorescence taken from a skin biopsy. He had a proteinuria at 1.8 g/24 h, and the renal biopsy concluded in a lupus nephritis of classes 5 and 3 with an activity index of 1. On the articular plan, he presented a chronic, non-deforming and non-destructive inflammatory polyarthritis. Hematologically, he had an autoimmune anemia (hemoglobin level 8.9 g/dL with positive Coombs test), and a lymphopenia at 960/mm3. Immunologically, the anti-nuclear and native anti-DNA antibodies were positive, and the C3 fraction of the complement was low. He had polyserositis including pleural and pericardial effusions. Indicated treatments were high-dose, long-term CS therapy (1 mg/kg/day) and hydroxychloroquine (HCQ), 400 mg/day.

**Fig. 1:**
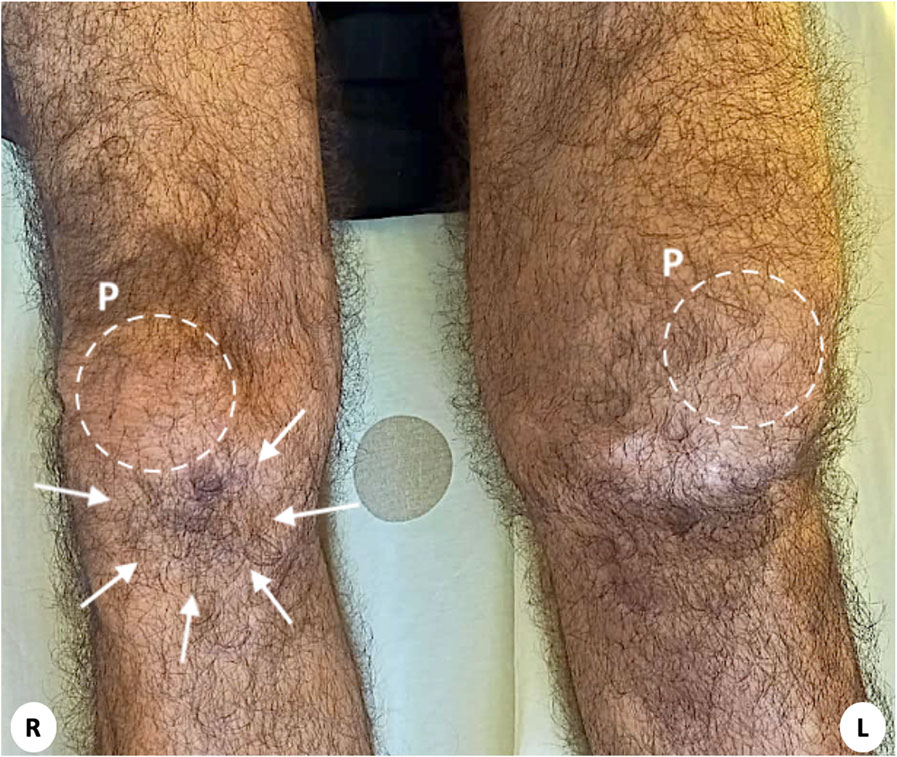
Clinical aspect of the knees showing a gap in the right infrapatellar region (arrows), an ascension of both patellae (P), and a swollen left knee

Two months after the start of treatment, the patient presented a painful buckling of both knees when walking, not responding to nonsteroidal anti-inflammatory drugs (NSAIDs). He developed total functional impotence 1 month later. When he consulted in our department, the patient was still on full-dose CS therapy, but had not started HCQ yet because he was scheduled for a complete ophthalmic check-up. He was unable to walk without crutches. The patellae were ascended, there was a gap in the right infrapatellar region, and the left knee was swollen (Fig. 1). Active knee extension was impossible.

**Fig. 2:**
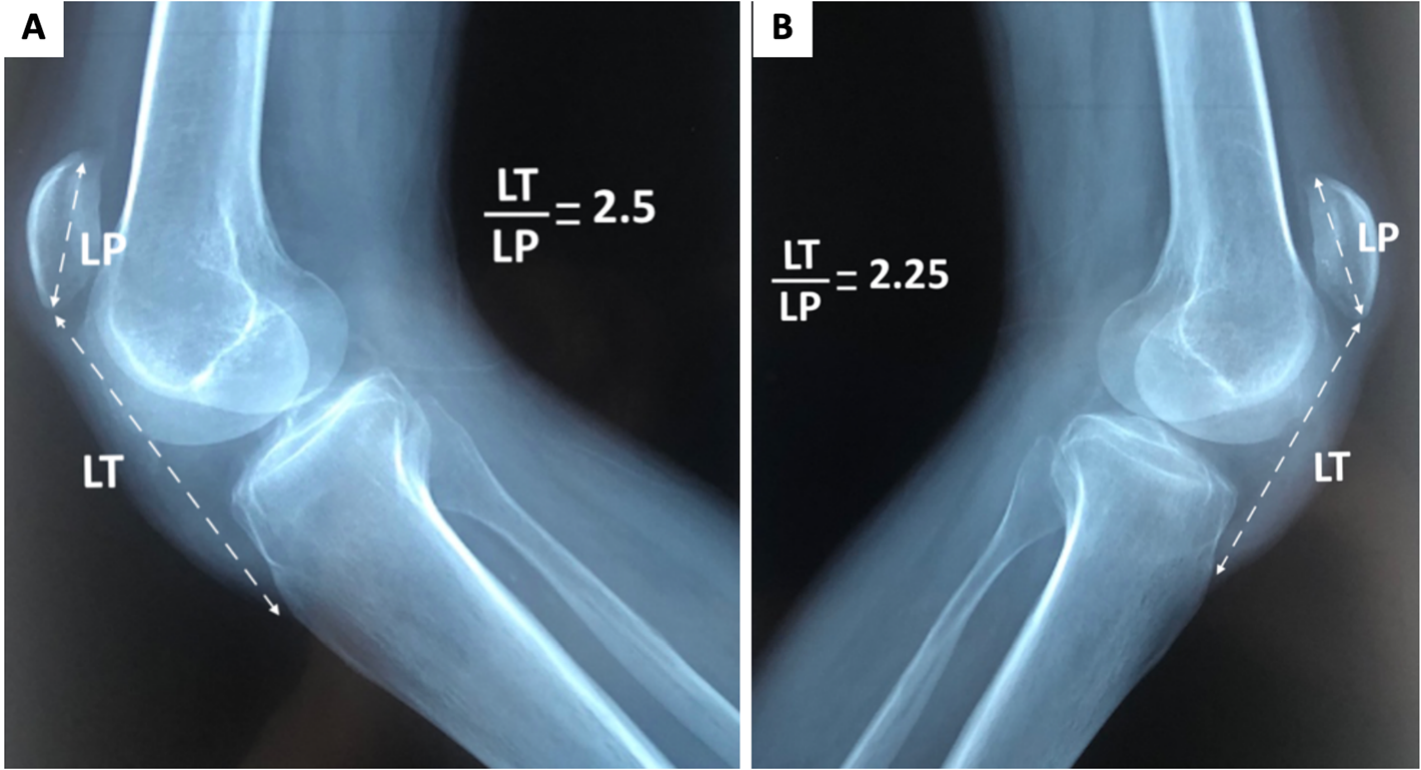
Conventional radiography findings in both knees (**a**: right knee, **b**: left knee). Bilateral aspect of patella alta with the Insall-Salvati ratio. LP: length of the patella; LT: length of the patellar tendon

**Fig. 3:**
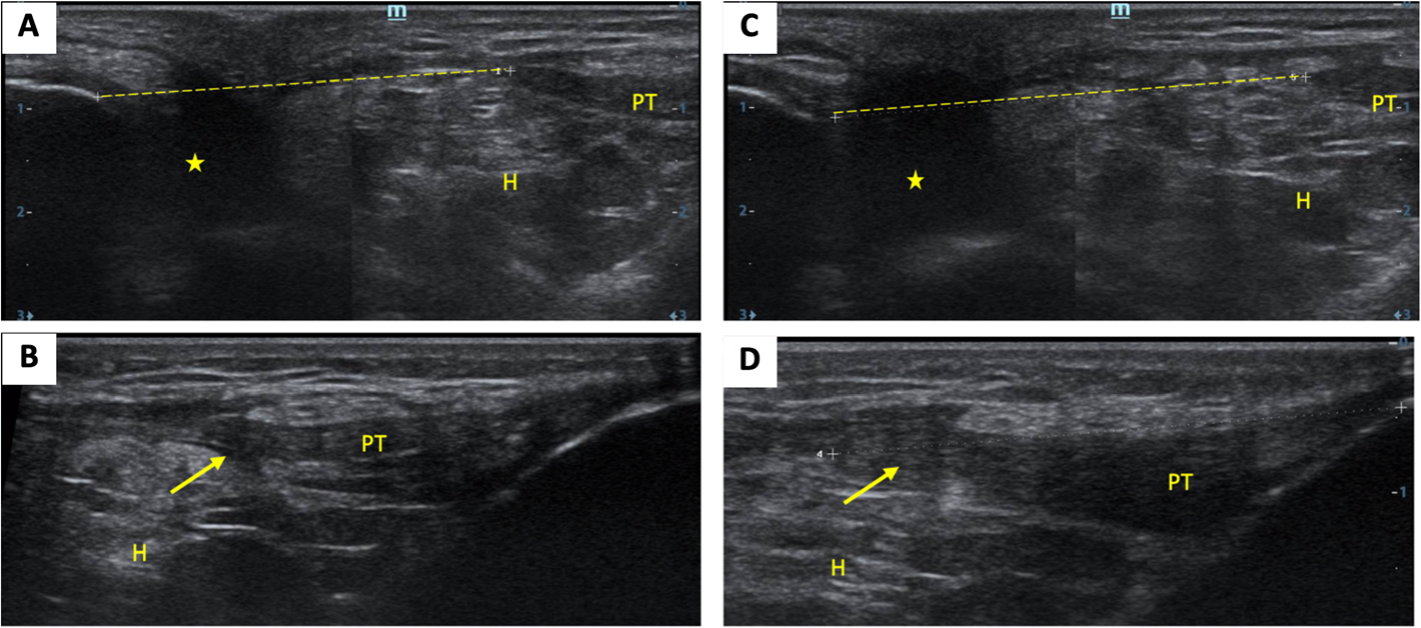
Ultrasound of the knees performed using a high frequency linear transducer. **a** and **b**: right knee. **c** and **d**: left knee. Median sagittal sections of the patellar tendons (PT) showing the stump of the patellar tendon (arrow) and the diastasis between the apex of the patella and the stump (discontinuous line), associated with an effusion of the suprapatellar bursa (yellow star) as well as an infiltration of Hoffa’s fat pad (H)

**Fig. 4:**
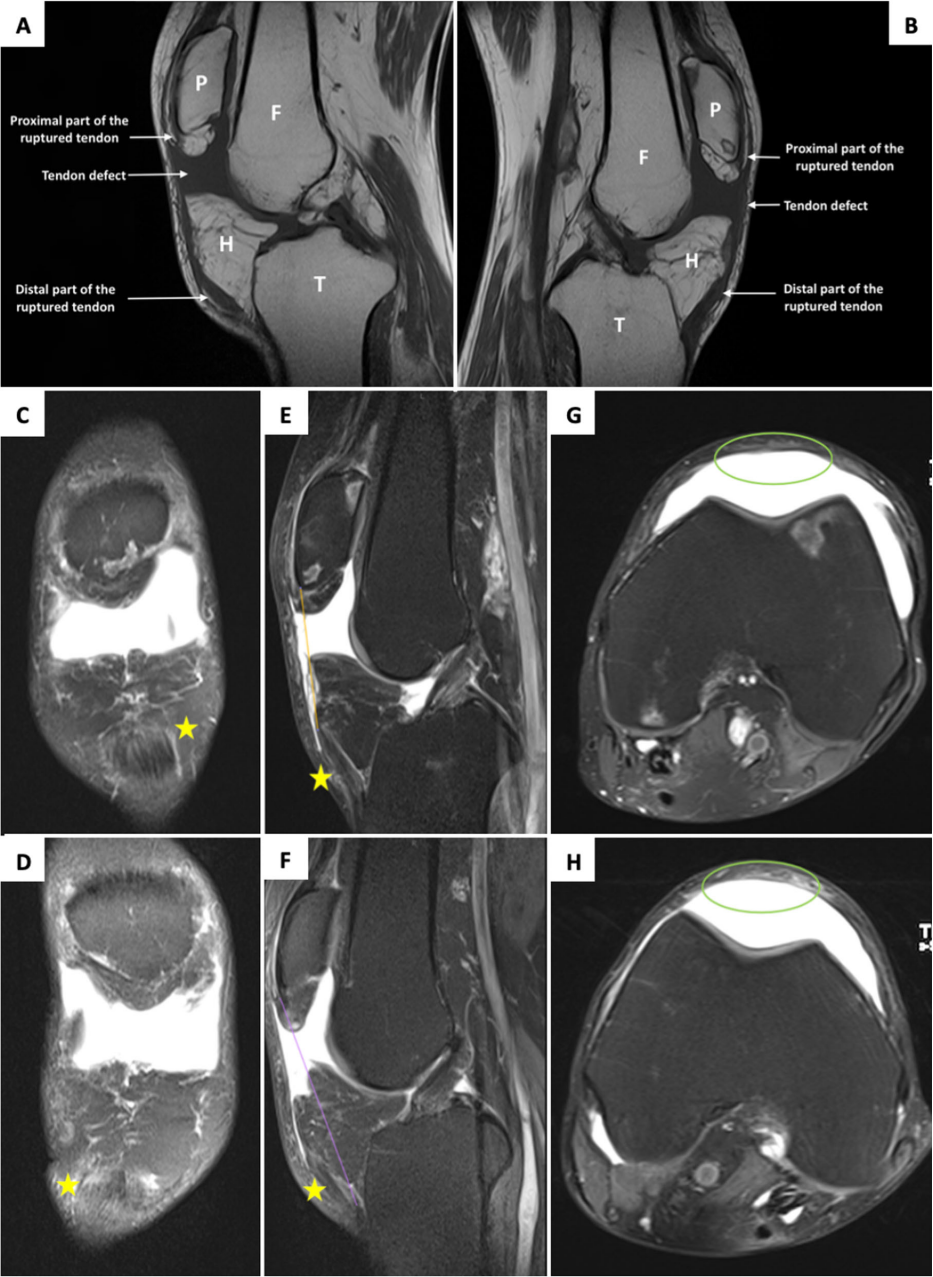
Sagittal T1-weighted MRI showing the rupture of the patellar tendon in the right (**a**) and left (**b**) knees (P: patella, F: femur, T: tibia, H: Hoffa’s fat pad). Proton Density Fat-Saturation MRI sequences (**c**: right coronal, **d**: left coronal, **e**: right sagittal, **f**: left sagittal, **g**: right axial, **h**: left axial) showing distal patellar tendons stumps (yellow stars), the diastasis and loss of substance of the patellar tendon (green ellipses)

The standard profile x-ray showed bilateral patella alta (PA), with an Insall-Salvati ratio (ISR) of 2.25 in the right knee and 2.2 in the left (normal range 0.8–1.2) (Fig. 2). MSUS revealed a complete rupture of the two patellar tendons (Fig. 3). Magnetic resonance imaging (MRI), indicated before surgery, had an ISR of 1.87 on the right side and 1.88 on the left side (normal range 0.74–1.5). The diastasis measured 40 mm on the right side and 45 mm on the left, filled on both sides with an effusion of great abundance (Fig. 4).

**Fig. 5:**
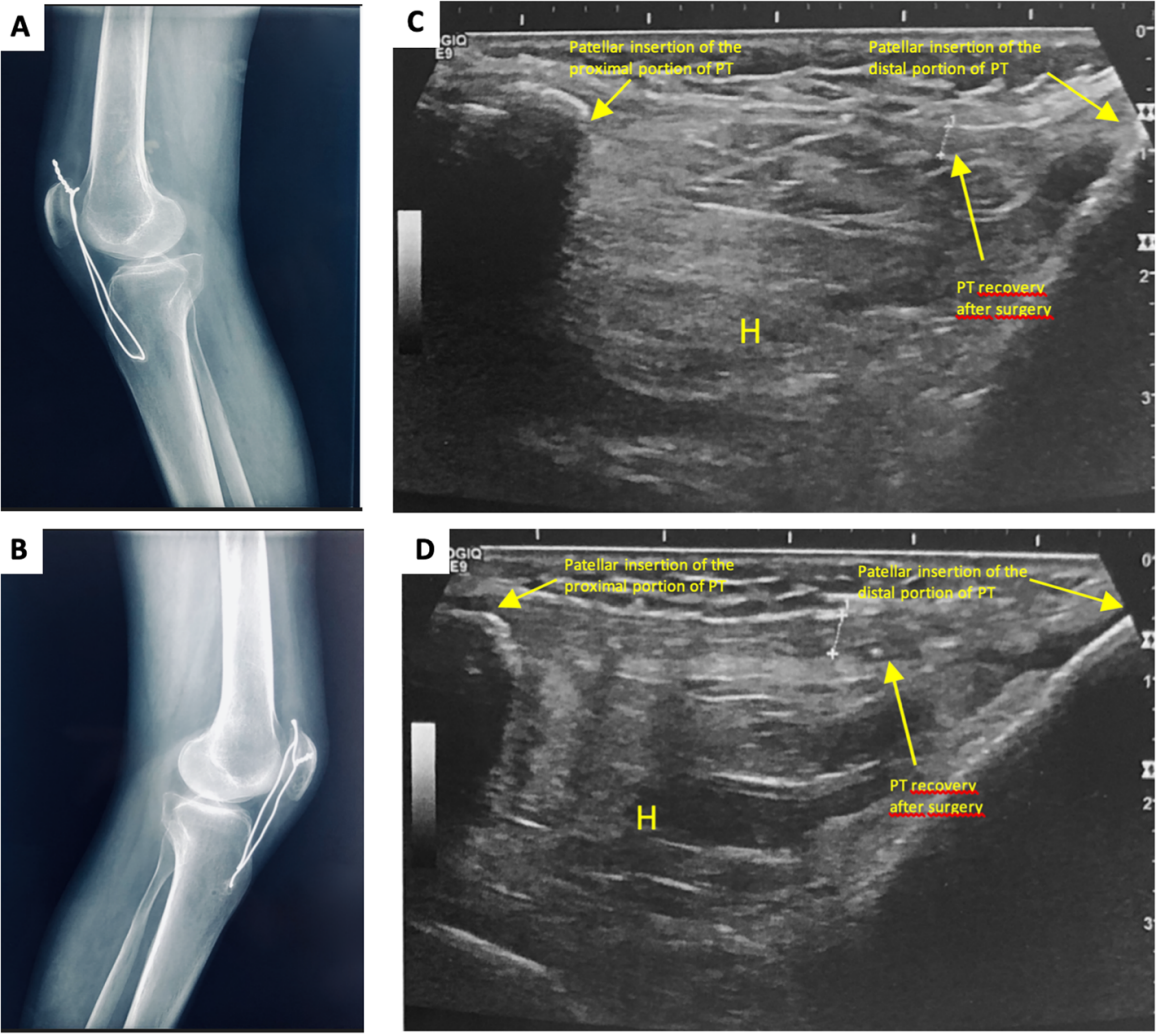
Control x-rays at 3 months showing the recovery of the patellar height with the cerclage wires in the right (**a**) and left (**b**) knees. Ultrasound longitudinal scans of the knees at 6 months of follow-up showing recovered patellar tendons in the right (**c**) and left (**d**) knees. PT: patellar tendon, H: Hoffa’s fat pad

The patient was referred to trauma surgeons. The underlying SLE was stable: laboratory blood tests showed an erythrocyte sedimentation rate of 13 mm, a C-reactive protein at 11.6 mg/L (normal < 5 mg/L). The surgery consisted in a repair of the patellar tendons with sutures, associated with a reinforcement wire cerclage in view of the poor tissue quality of the tendons. There were no postoperative complications. Physiotherapy was undertaken after immobilization by removable braces for 4 weeks. Sessions consisted in progressive extension/flexion movements, and walking rehabilitation using a walking frame. After 2 weeks the patient was able to perform 80° flexion of the right knee and 60° of the left. He was dispensed with the walking frame, replaced by a single forearm crutch after 1 month of rehabilitation. The 3-month follow-up x-ray showed patellae in their normal position (Fig. 5, a and b). At 6 months postoperatively, the range of motion was 0°–120° of flexion in the right knee and 0°–110° in the left. He was able to mobilize independently. The SLE was stable under 15 mg/day of CS and HCQ 400 mg/day. The control MSUS showed the stability of the surgical repair (Fig. 5, c and d).

## Discussion and conclusion

This is a case of BPTR in a patient with SLE, happening 2 months after the start of oral full-dose CS, and with a recovery of knee function after surgery and rehabilitation.

Tendon rupture usually is the result of underlying tendinosis or a weakened tendon by chronic inflammatory diseases, renal dialysis, patellar tendinopathy or previous injury [1]. Patellar tendon rupture is the third most frequent cause of disruption of the knee extensor mechanism after patella fractures and quadriceps tendon rupture [3], with an incidence of 0.5% in the United States [1]. BPTR is much rarer, with sporadic cases in the literature [4]. It has been reported in SLE [3], rheumatoid arthritis [5], chronic renal failure [6], and primary hyperparathyroidism [7]. Moreover, cases have been reported in apparently healthy people [8], and during sports activities [9]. Treatments like CS [10], FQ [11] and statins [12] also predispose to BPTR.

There are 3 categories of pathophysiological mechanisms being responsible for BPTR according to Taylor et al. [2]. First, the involvement of systemic or autoimmune diseases, causing inflammatory reactions, and weakening the tendon structure, resulting histologically in chronic inflammation and amyloid deposition. Second, the use of treatments such as oral or injectable CS, weakening the tendon by affecting the synthesis of collagen. Other incriminated treatments are FQ, which decrease fibroblasts proliferation [13], and statins, causing gross changes in the organization of collagen fibers and thinning of the epitenon [12]. The third pathophysiological category includes repeated microtraumas, causing degenerative tendon damage [2]. Our patient presented two of these risk factors: the underlying SLE, and the treatment with long-term CS.

In case of BPTR, both knees present symmetrical physical signs: swelling, ascending patellae with gaps in the infrapatellar regions. In our case, only the left knee was swollen, and only the right knee had a visible gap. This difference could have led to a diagnosis delay for our patient, who was prescribed NSAIDs without improvement. Siwek and Rao reported that 28% of their BPTR patients were not diagnosed during their initial examination [14]. This highlights the non-specific clinical features of BPTR. Consequently, health care providers must be aware of this condition and of its risk factors in order to prevent diagnosis delay. Knee extension deficit should have the patellae carefully examined.

Radiologically, standard profile x-rays on 30° flexed knees aim to assess the aberrant high position of the patellae, called patella alta, which is quantified by the ISR [15]. It consists in calculating the ratio of the length of the patellar tendon (from the lower pole of the patella to its insertion on the tibia) over the patellar length (greatest pole-to-pole length). The normal range is 0.8–1.2, and the diagnosis of PA is made if the ISR is greater than 1.2. In our patient, this ratio was 2.5 in the right knee and 2.25 in the left. Standard x-ray diagnoses PA, but does not specify its etiology, which is multiple: patellar tendon rupture, subluxation of the patella, Sinding-Larsen-Johansson disease, and joint effusion [16]. This highlights the interest of MSUS, which is a simple, easy to access, fast, and cost-effective imaging method of joint structures [16]. In case of a complete tendon rupture, the image is a total interruption of the normal fibrillar structure, with a continuity solution [16], which was found in our patient. MRI has an interest mainly for surgery: in addition to confirming the PA with a better assessment of the ISR, it provides more details about the topography and detects associated lesions.

The most often performed procedure is tendon repair by sutures on a healthy tendon [14]. In case of tendon fragility, the use of wire cerclage reduces the tension on the suture line and the risk of secondary rupture [17]. Our patient had an additional wire cerclage because the risk of secondary rupture is constant: chronicity of the SLE, and long-term CS therapy. Furthermore, Siwek and Rao insist on early diagnosis and prompt surgical repair to avoid tendon retraction and surgical failure [14].

In conclusion, BPTR is a rare complication of SLE and long-term CS therapy. The inflammatory reactions of the systemic disease, and the lack of collagen synthesis due to CS therapy, are among trigger factors. This condition should be suspected in a patient unable to perform active knee extension, and presenting bilateral PA. MSUS provides a rapid and reliable diagnosis. Early assessment and treatment give a better functional prognosis.

## Data Availability

Data concerning the patient’s record are available from the corresponding author on reasonable request.

## Abbreviations

BPTR: Bilateral patellar tendon rupture
CS: Corticosteroids
FQ: Fluoroquinolones
HCQ: Hydroxychloroquine
ISR: Insall-Salvati ratio
MRI: Magnetic resonance imaging
MSUS: Musculoskeletal ultrasound
PA: Patella Alta
SLE: Systemic lupus erythematosus
